# Bilateral temporal lobe disease: looking beyond herpes encephalitis

**DOI:** 10.1007/s13244-016-0481-x

**Published:** 2016-02-24

**Authors:** Ayelet Eran, Adina Hodes, Izlem Izbudak

**Affiliations:** Department of Radiology, Neuroradiology Unit – Rambam Health Care Campus, P.O.B 9602, Haifa, 31096 Israel; Technion, Israel Institute of technology, Haifa, Israel; Department of Radiology, Johns Hopkins Medical Institutions, Baltimore, MD USA

**Keywords:** Temporal lobes, Herpes encephalitis, Dementia syndromes, Posterior circulation brain infarction, Temporal lobe epilepsy

## Abstract

**Abstract:**

The temporal lobes have unique architecture, and functionality that makes them vulnerable to certain disease processes. Patients presenting with bilateral temporal lobe disease are often confused and have altered consciousness, and are therefore unable to provide cogent histories. For these reasons, imaging plays an important role in their workup and management. Disease entities causing bilateral temporal lobe involvement can be infectious, metabolic, neoplastic, and degenerative aetiologies, as well as trauma and cerebrovascular events. We will first describe the structural and functional anatomy of the temporal lobes and explain the mechanisms that underlie bilateral temporal lobe disease, and then show and discuss the different disease entities and differential diagnosis.

***Teaching points*:**

• *Bilateral temporal lobe disease is a unique pattern with specific differential diagnosis*.

• *Patients presenting with bilateral temporal lobe disease are often confused*.

• *Radiologists should be familar with the variety of disease processes that cause bitemporal disease*.

## Temporal lobe anatomy

**Fig. 1:**
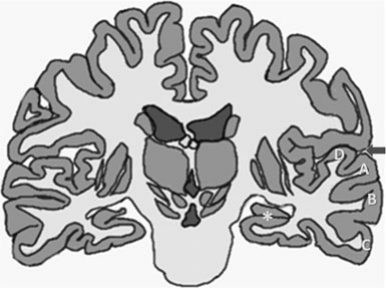
Schematic drawing of the brain in coronal section. Superficially, the superior, middle and inferior temporal gyri (**a**, **b**, **c** respectively) comprise the temporal lobe. The sylvian fissure (*arrow*) demarcates the border with the frontal lobe. The primary auditory cortex is located in the transverse temporal gyrus (of Heschl) (**d**). The parahippocampal gyrus and hippocampus (*asterisk*) lie in the medial aspect of the temporal lobe. The hippocampus runs superior and lateral to the parahippocampal gyrus from rostral to caudal

Functional centres of hearing, speech, memory, olfaction, sensation, emotion and behaviour are located in the temporal lobes, which lie in the middle cranial fossa, lateral to the midbrain and inferior to the basal ganglia. Each temporal lobe is separated from the frontal lobe and the anterior parietal lobe by the Sylvian fissure (Fig. 1). On axial images along the canthomeatal plane, the temporal lobes do not extend above the level of the lateral ventricles [1].

**Fig. 2:**
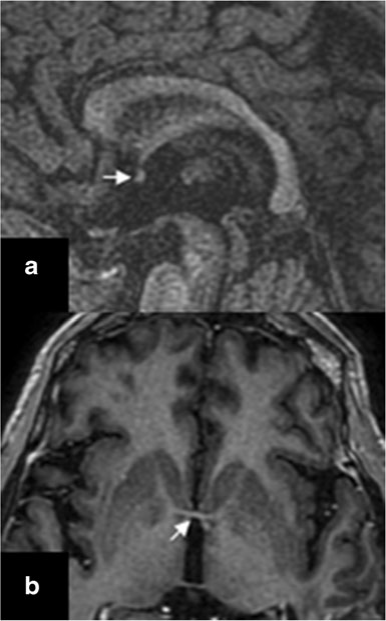
Sagittal (**a**) and axial (**b**) T1W images showing the anterior commissure (*arrows*)

Although the temporal lobes are not situated one next to each other, they are interconnected through the anterior commissure, the corpus callosum, and the hippocampal commissure. Those connections are one of the mechanisms that contribute to bilateral temporal lobe disease. The anterior commissure is a white matter commissural tract that crosses the midline in the anterior wall of the third ventricle, at the junction of the lamina terminalis and the rostrum of the corpus callosum [2, 3]. Given its latero-lateral orientation, the commissural tract is readily visible on axial and sagittal planes (Fig. 2). The corpus callosum consists of nerve fibres interconnecting the brain hemispheres. The fibres that connect the temporal lobes cross midline within the splenium and mid-body of the corpus callosum [4]. The hippocampal commissure is a commissural tract that carries decussating fibres from the forniceal crura; it crosses the midline below the undersurface of the body of the corpus callosum. The optic tract and radiation may spread pathology from the optic chiasm to both temporal lobes.

The limbic system is a set of medially located supratentorial brain structures that constitute a system controlling emotion, behaviour and memory, as well as neuroendocrine and autonomic functions [2]. The hippocampus and parahippocampal gyrus are parts of the limbic system situated within the temporal lobe (Fig. 1). Some disease processes have selective affinity to temporal lobe due to selective limbic system vulnerability, which might be immune mediated, as in limbic encephalitis [5]. Affinity to the allocortical elements of the temporal lobe as occur in Alzheimer’s disease [6], and sensitivity to hypoxia of the amygdala and hippocampus also contribute to selective temporal lobe disease.

Arterial supply to the temporal lobes is derived from both the anterior and posterior circulation. Inferior branches of the middle cerebral artery (MCA) supply the lateral aspect of the temporal lobe, the anterior choroidal arteries supply the uncus and the piriform gyrus and temporal branches of the posterior cerebral artery (PCA) supply the medial aspect of the temporal lobe and pes hippocampi [1]; therefore, the tip of a basilar artery stroke can manifest as bilateral temporal lobe injury.

In the following, we show and demonstrate the different disease entities causing bilateral temporal lobe disease and discuss ways to differentiate between them.

## Infectious diseases

### Herpes encephalitis

**Fig. 3:**
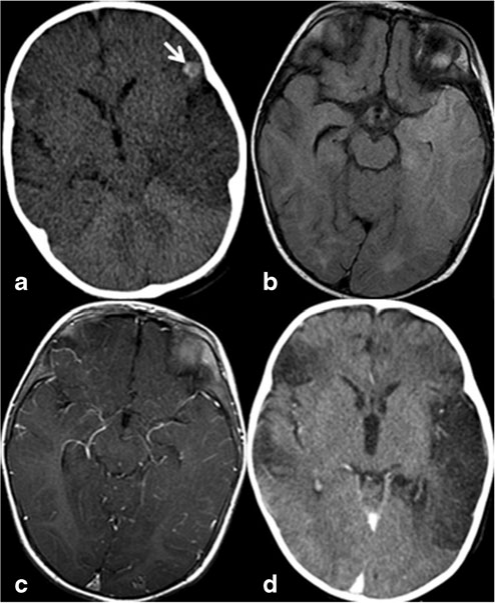
A 7-month-old infant with herpes encephalitis. At presentation (**a**), asymmetric hypodensity is noted in the left temporal lobe on noncontrast CT and subtle hypodensity in the right temporal lobe. Hemorrhagic focus is noted on the left frontal operculum (*arrow*). MRI at presentation shows bilateral temporal lobe FLAIR hyperintensity left greater than right (**b**) and leptomeningeal enhancement (**c**). Delayed contrast-enhanced CT (**d**) shows development of encephalomalacic changes in the temporal lobes bilaterally, left greater than right and in the right frontal lobe

Herpes encephalitis is the most common and gravest form of acute encephalitis, and is almost always caused by latent Herpes Simplex Virus-1 (HSV-1). The latent virus spreads retrograde from the trigeminal ganglion, located at the medial aspect of the temporal fossa, along trigeminal nerve fibres innervating the leptomeninges of the anterior and middle cranial fossa. Patients present acutely with fever, headaches, seizures and altered mental status. Bilateral temporal lobe involvement is usually asymmetric, accompanied by insular disease, and stops at the lateral putaminal border. CT may be normal or show oedema. On MRI, increased T2 signal involving cortex and white matter of the temporal lobes and gyral enhancement are typical. Cortical microbleeds may be seen on T2* imaging [7]. Other brain regions might be involved, most commonly the cingulum of the frontal lobes (Fig. 3).

### Human herpes virus 6 encephalopathy

**Fig. 4:**
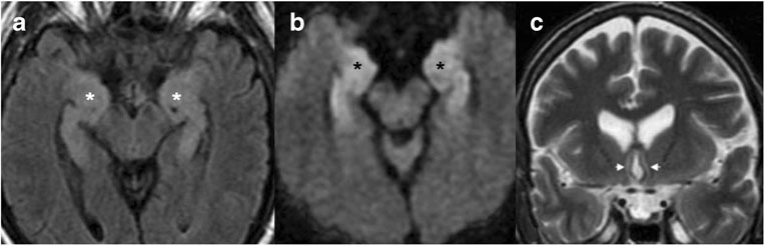
**a**–**c** A 66-year-old male with confusion 10 days after allogeneic bone marrow transplantation. Increased T2/FLAIR signal and restricted diffusivity are noted in the mesial temporal lobes (*asterisks* in **a**, **b**), and forniceal columns (*arrowheads* in **c**), compatible with HHV 6 encephalopathy

Human herpes virus 6 (HHV-6) is a neurotrophic virus, known to cause roseola infantum and exanthem subitum in childhood. More than 90 % of the population by the age of 2 years is seropositive to the virus and most infections are asymptomatic [7]. The virus can reactivate and cause encephalopathy in immunocompromised hosts, mostly patients undergoing bone marrow and solid organ transplantation (Fig. 4). Patients will present about 3 weeks post transplantation with change in mental status, loss of short-term memory and seizures. CT is often normal and MRI shows increased T2/FLAIR and DWI signal, usually with no enhancement or haemorrhage in mesial temporal lobes, with possible involvement of additional limbic system components [7].

Although herpes simplex encephalitis and HHV-6 encephalitis can be discerned clinically, the following imaging features are more common in herpes simplex encephalitis: (1) CT abnormalities in the early phase of the disease, (2) extra-temporal disease and asymmetric distribution, (3) signal abnormalities in the subacute/chronic phase [7].

### Fungal infection

**Fig. 5:**
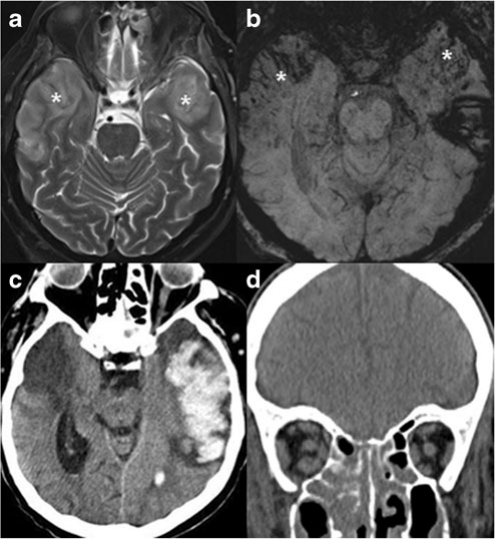
Mucormycosis infection of the temporal lobes bilaterally in a 62-year-old patient, immuncomprmised due to steroid treatment. Increased T2 signal (**a**) and blood products (**b**, SWI) are noted in the anterolateral temporal lobes (*asterisks*). The patient later developed massive left temporal lobar haemorrhages (**c**), and deceased. On coronal CT image of the same patient (**d**), note partial opacification of the ethmoid and maxillary sinuses and stranding of the intra-orbital fat. Autopsy revealed mucormycosis in brain parenchyma and paranasal sinuses

Fungal infections such as mucormycosis may spread from the maxillary and ethmoid sinuses across skull base to the temporal lobes in immune compromised patients. Due to the direct spread, brain disease is usually the diffuse cerebral parenchymal type, with brain oedema, haemorrhages and irregular enhancement (Fig. 5) [8]. In cases of immune deficiency and paranasal sinuses infection, it is crucial to carefully inspect the orbits, deep facial planes of the upper neck and intra-cranial content, and to look for signs of fungal infection and extra-sinus disease.

Direct spread of an infection to the temporal lobe might also occur from bilateral otomastoiditis.

Syphilis and flavivirus are additional infections agents reported to cause preferential bilateral temporal lobe disease [9, 10].

## Inflammation

### Limbic encephalitis

Limbic encephalitis is a family of disorders causing inflammation of components of the limbic system; it can be paraneoplastic and non-paraneoplastic.

**Fig. 6:**
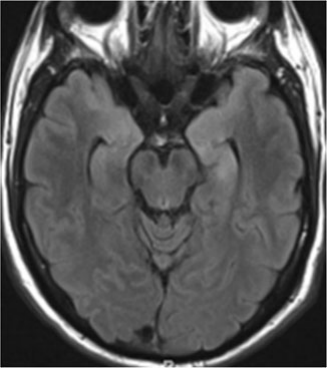
A 52-year-old male with known diagnosis of small cell lung carcinoma and new onset seizures. Increased FLAIR signal is noted in the mesial temporal lobes, left greater than right without mass effect or volume loss. Based on positive serum anti Hu antibodies and imaging findings, the diagnosis of paraneoplastic limbic encephalitis was made

Paraneoplastic limbic encephalitis is immune-mediated encephalitis, likely resulting from the immune response to an antigen shared by a neoplasm and the nervous system [5]. Patients usually present subacutely with personality changes, irritability, depression, dementia, seizures and short-term memory loss. It is mostly associated with small cell lung carcinoma and testicular germ cell tumour, and may be diagnosed up to 4 years before tumour diagnosis (Fig. 6). Anti-neuronal antibodies are found in about 60 % of the patients (anti Hu, anti Ta, anti Ma, etc.). The diagnosis requires the combination of clinical, imaging and laboratory tests, as well as exclusion of other neuro-oncologic conditions.

Imaging abnormalities are seen in 64 % of the patients on MRI, most commonly, unilateral or bilateral mesio-temporal T2/FLAIR signal abnormalities. Enhancement may occur, as well as involvement of additional limbic system components and other brain regions [5].

Non-paraneoplastic limbic encephalopathy is a similarly rare condition, without an underlying malignancy [11]. Imaging findings are similar to neoplastic limbic encephalitis; clinically, seizures and prolonged presentation are more common. Diagnosis relies mainly on exclusion of an underlying malignancy.

## Neurodegenerative diseases

### Alzheimer disease

**Fig. 7:**
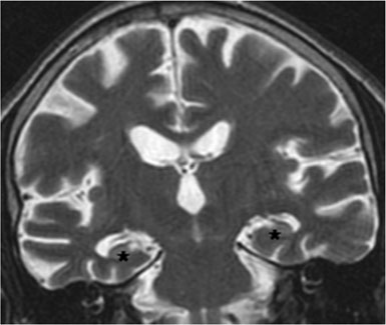
A 68-year-old male with clinical diagnosis of sporadic Alzheimer disease. Coronal T2W image shows diffuse mild to moderate brain parenchymal volume loss and bilateral hippocampal atrophy, right greater than left (*asterisks*)

Alzheimer disease is the most common type of dementia, characterized by cognitive impairment severe enough to interfere with daily living. Bi-temporal involvement is a known feature (Fig. 7), with disproportional volume loss in the mesial temporal structures, especially the entorhinal cortex [12]. This is thought to be the consequence of increased vulnerability of the hippocampal formation and entorhinal cortex to neuritic plaque deposition and subsequent damage.

**Fig. 8:**
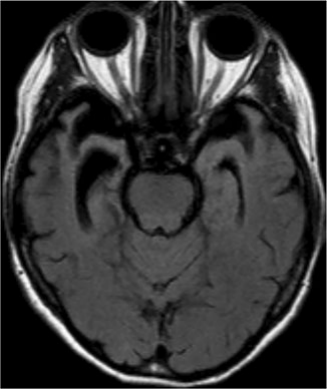
A 63-year-old female who presented with 2 years history of cognitive decline, mainly in semantic and visual recognition functions. On axial FLAIR image, there is asymmetric temporal lobe atrophy without gliosis, right greater than left. Based on cognitive tests, clinical presentation and imaging findings the diagnosis of semantic type of fronto-temporal dementia was made. This pattern of atrophy should be distinguished from post-traumatic encephalomalacia following bilateral temporal contusions, based on clinical history and the absence of gliosis and blood byproducts on imaging

Frontotemporal dementia is the second most common type of presenile dementia, and encompasses a heterogeneous group of disorders with preferential temporal and frontal atrophy [13]. Preferential and usually asymmetric temporal lobe atrophy is seen in the semantic variant of this syndrome (Fig. 8); otherwise, atrophy frequently extends beyond the temporal lobe.

The two diseases are usually differentiated based on clinical assessment and cognitive tests, with structural imaging serving as a supporting and follow-up tool [14].

## Metabolic diseases

**Fig. 9:**
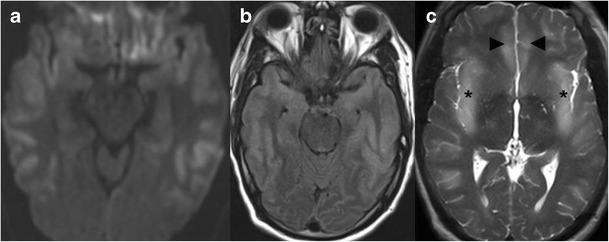
A 42-year-old male with adult onset ornithine transcarbamylase deficiency who presented with acute confusional state. Restricted diffusivity (**a**) and increased FLAIR signal (**b**) in the temporal lobes, as well as increased T2 signal in insular and cingulate gyri (**c**, *asterisks* and *arrowheads*, respectively). Note symmetrical distribution and prominent cortical involvement, as opposed to Fig. 3, which shows Herpes encephalitis

Selective temporal lobe involvement can be seen in adult onset ornithine transcarbamylase (OTC) deficiency (Fig. 9). In this milder form of the x-linked disease, patient present later in life (between infancy to adulthood) with signs of encephalopathy. MRI shows swelling, increased T2 signal and diffusion restriction without enhancement mainly in the insular and cingulate cortex in a symmetric distribution. The selective injury is thought to result from increased vulnerability to hyperammonemic hyperglutaminergic status [15]. The clinical and imaging characteristics of this disease should be differentiated from Herpes encephalitis. The symmetrical distribution and lack of blood byproducts and enhancement are helpful imaging clues.

Preferential hippocampal damage may result from hypoxic ischemic insult [16]. Carbon monoxide toxicity is known to damage Ammon’s horn cells; usually this will be accompanied by injury to the additional vulnerable brain regions.

## Epileptogenic syndrome

**Fig. 10:**
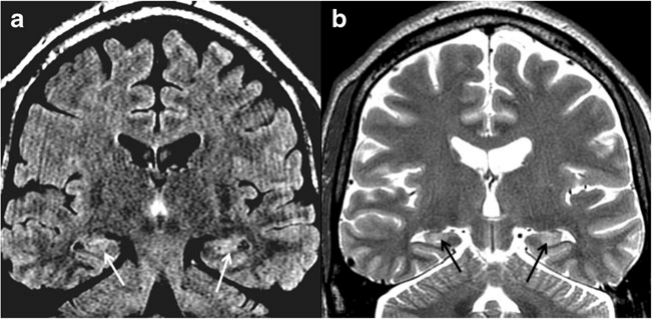
A 27-year-old female with persistent partial complex seizures. On coronal FLAIR (**a**) and T2 images, there is bilateral hippocampal atrophy and increased T2/FLAIR signal (arrows), compatible with bilateral mesial temporal sclerosis. Note mild diffuse atrophy possibly related to chronic epilepsy and drug effect

Mesial temporal sclerosis is the most common pathological substrate for partial complex seizures. The disorder results from idiopathic neuronal loss and gliosis, which are visualized on MRI as increased T2/FLAIR signal, loss of the internal hippocampal architecture and atrophy. These may also involve the ipsilateral fornix, which carries afferent and efferent hippocampal fibres, mammillary bodies and parahippocampal gyrus [17]. Although the disease is usually unilateral, it can be bilateral in up to 10 % of cases (Fig. 10) [18]. High resolution oblique coronal thin sections T2/FLAIR MRI images of the temporal lobes perpendicular to the temporal horn are highly sensitive for depicting hippocampal abnormalities.

Limbic encephalitis also commonly manifest with seizures. The extensive limbic system involvement, lack of atrophy, presence of enhancement and extra-temporal lobe disease are possible features of limbic encephalitis and not mesial temporal sclerosis. Mesial temporal sclerosis should also be differentiated from other temporal lobe epileptogenic diseases, but this is beyond the scope of this review (see Ref. 17 for additional reading).

## Neoplasm

### Gliomas

Neoplasms can spread from one temporal lobe to another through the previously mentioned white matter tracts interconnecting the temporal lobes. This pattern of spread is most commonly seen in glial cell tumours, specifically astrocytomas, which are the most common gliomas. Astrocytomas can be graded from low-grade circumscribed neoplasm, through diffuse astrocytoma, anaplastic astrocytoma and glioblastoma multiforme, which is a World Health Organisation (WHO) grade IV tumour. On imaging, in general, the degree of enhancement and oedema increase with increasing tumoral grade [19].

**Fig. 11:**
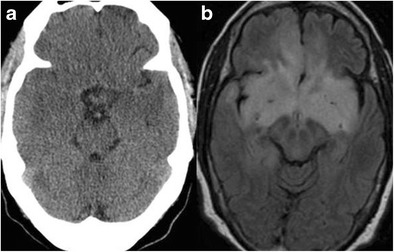
A 42-year-old female with new onset seizures. On noncontrast CT (**a**), only mild mass effect is noted on the Sylvian fissure and subtle symmetric hypodensity in the temporal lobes. On MR (**b**), the abnormality is clearly visualized as bilateral temporal and insular FLAIR hyperintensity, crossing midline along the anterior commissure and also extending to the frontal lobe. The lesion did not enhance and biopsy confirmed the diagnosis of low-grade glioma

Anterior spread along the anterior commissure is the most common pattern of bilateral temporal lobe disease (Fig. 11). Posteriorly, spread is through the corpus callosum; however preferential isolated infiltration of the temporal lobes through this connection is unlikely.

**Fig. 12:**
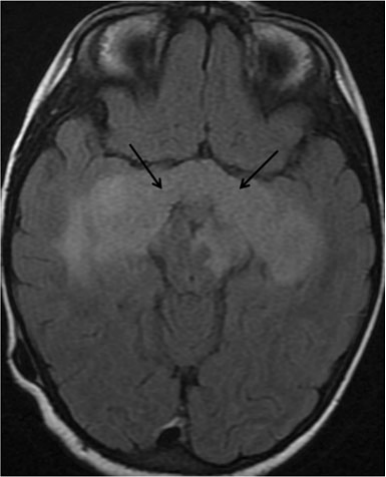
A 3-year-old child with visual difficulties. Axial FLAIR image show a supra-sellar lesion extending to the temporal lobes along the optic tracts (*arrows*) with moderate mass effect, compatible with optic glioma. FLAIR hyperintensity is also noted in the left mesencephalon from additional tumoral involvement

Optic nerve glioma may also spread to both temporal lobes from the optic chiasm along the optic tracts and radiation (Fig. 12).

Neoplasms usually present with an insidious course of worsening neurological deficits or new onset seizures; however, in the extreme case of herniation or significant mass effect, the patient may arrive comatose, and imaging will play an important role in treatment decision. The main concern will be to differentiate herpes encephalitis from a tumour. Features supporting the diagnosis of a tumour will be spread along nerve tracts and parenchymal instead of cortical enhancement.

## Cerebrovascular disease

**Fig. 13:**
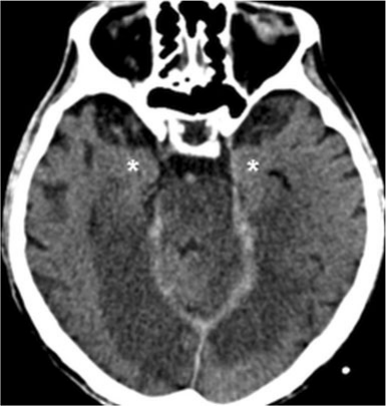
An 83-year-old male who presented with acute coma. Noncontrast CT obtained 2 days after admission shows hypodensity in the medial aspects of both temporal lobes, compatible with posterior circulation ischemia. Occipital lobes, brainstem and cerebellum are also involved, and there is relative hyperdensity of the basilar artery . Note preservation of the entorhinal cortex (*asterisks*), supplied by MCA and anterior choroidal artery branches

Basilar tip and bilateral PCA occlusion cause ischemic injury in the medial aspect of both temporal lobes and/or the midbrain and thalamus. The patient presents acutely with visual and memory deficits and altered mental status. Brainstem, cerebellar and thalamic dysfunction may also be present. CT and MR demonstrate ischemic lesions in the medial aspect of both temporal lobe as well as occipital lobes (Fig. 13). Lesions’ distribution and diffusion restriction as well as acute presentation may resemble those of herpes encephalitis and adult onset OTC deficiency. Involvement of grey and white matter, and absence of early enhancement and vascular distribution of the lesions support the diagnosis of ischemia as well as demonstration of occluded vessel on angiographic study.

In CADASIL (Cerebral Autosomal Dominant Arteriopathy with Subcortical Infarcts and Leukoencephalopathy), focal lesions of increased T2 signal are seen in the periventricular and deep white matter throughout the brain, as well as lacunar infarctions; however, anterior temporal lobe and external capsule involvement are typical [20]. Subcortical white matter lesions in the temporal lobes are also seen in patients with myotonic dystrophy [21].

## Trauma

**Fig. 14:**
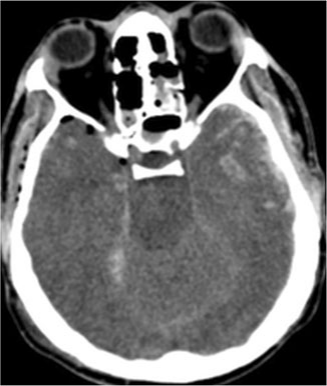
A 19-year-old male, following a high energy motor vehicle accident with hemorrhagic contusions in the temporal lobes bilaterally. Note pneumocephalus anterior to the right temporal lobe, small amount of subarachnoid blood along the right tentorial leaf and subarachnoid blood along the left temporal lobe. Additionally, there is relative sulcal effacement and loss of grey-white matter differentiation from diffuse brain swelling

The anterior temporal lobes are susceptible to traumatic injury due to deceleration forces compressing the temporal tip against the sphenoid wing. Hemorrhagic cortical contusions are the most common injury (Fig. 14). Subdural hematomas may also occur.

## Radiation necrosis

Radiation necrosis may develop in both temporal lobes following radiation of head and neck malignancy, usually nasopharyngeal carcinoma [22]. Familiarity with radiation ports and patients history will facilitate diagnosis.

**Fig. 15:**
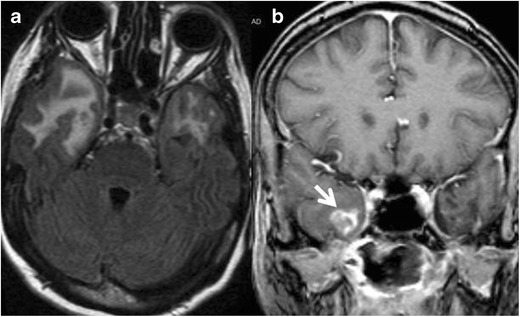
A 42-year-old female with a history of radiation for nasopharyngeal carcinoma. Axial FLAIR image (**a**) shows vasogenic edema in the anterior aspect of the temporal lobes bilaterally, right greater than left. Coronal contrasted T1 weighted image (**b**) shows enhancement on the right (*arrow*). On biopsy, the diagnosis of radiation necrosis was confirmed

MRI will show oedema and enhancement that is typically cortical (Fig. 15). In questionable cases, diffusion-weighted imaging, perfusion MR, or PET can be helpful for further evaluation [23].

## Mimickers

On CT, beam hardening artefacts may obscure or mimic bilateral temporal lobe disease.

**Fig. 16:**
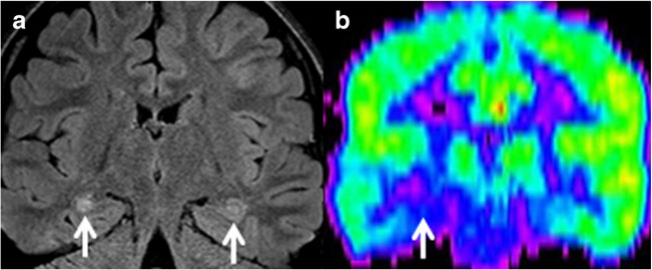
(**a**) Coronal FLAIR image of a 31-year-old man with partial complex seizures showing bilateral FLAIR hyperintensities in hippocampal body, right greater than left (*arrows*), suspected for bilateral mesial sclerosis. PET study (**b**), show unilateral hypometabolic focus (*arrow*), compatible with right-sided mesial temporal sclerosis

**Fig. 17:**
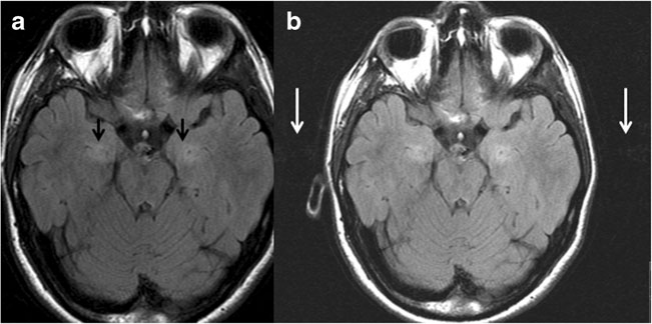
A 25-year-old man referred to MRI due to headaches. On axial FLAIR images, there is suspicious hyperintensity in the medial temporal lobes without atrophy or mass effect (*black arrows*). The abnormality was not enhancing and was not clearly seen on sagittal T2 weighted images. By using a wider window, repetitive ghosting is seen outside the anatomy along the phase encoding direction, compatible with pulsation artifact

On MRI, the relative normal T2 hyperintensity of the medial temporal lobes may mimic a lesion (Fig. 16). Pulsation artefacts from the carotid siphon may also mimic temporal lobe lesions (Fig. 17).

## Conclusion

The temporal lobes may be preferentially diseased in a variety of clinical scenario. In Table 1, we summarize the disease processes presented in this manuscript, with their typical clinical and imaging presentations and appropriate differential diagnoses. Familiarity with those diseases should assist prompt diagnosis that relies on the integration of clinical and imaging data.

**Table 1:** Summary of common disease processes causing bilateral temporal lobe disease

Disease	Main clinical characteristics	Main imaging characteristics	Main differential diagnosis
Herpes encephalitis	Fever, headaches, seizures and altered mental status	Asymmetric bilateral temporal lobe disease; insular disease; stops at the lateral putaminal border. Gyral enhancement and cortical microbleeds on MRI	HHV-6 encephalopathy, fungal infection, adult type OTC deficiency, glioma
HHV-6^*^ encephalop athy	Change in mental status, loss of short-term memory and seizures about 3 weeks post-transplant	Symmetric mesial temporal lobe and limbic system disease	Herpes encephalitis, limbic encephalitis
Fungal infection	Spread of paranasal sinus infection in immune-compromised patients	Brain edema, hemorrhages, irregular enhancement. Paranasal sinus infection	Herpes encephalitis
Limbic encephalitis	Subacute presentation with personality changes, irritability, depression, dementia, seizures and short-term memory loss	Unilateral or bilateral mesio-temporal disease. Enhancement may occur as well as involvement of additional limbic system components and other brain regions	HHV-6 encephalopathy, Alzheimer disease, Fronto-temporal dementia
Alzheimer disease	Cognitive decline interfering with daily activities	Disproportional asymmetric volume loss in the mesial temporal structures	Fronto-temporal dementia, limbic encephalitis, remote insult (sequel of herpes encephalitis, trauma)
Fronto-temporal dementia	Changes in personality and behavior and decline in semantic performance	Asymmetric temporal lobe atrophy in semantic variant of the disease	Alzheimer disease, remote insult (sequel of herpes encephalitis, trauma)
Adult type OTC^†^ deficiency	Acute encephalopathy	Symmetric disease of the insula and cingulate gyrus with increased T2 signal and diffusion restriction, and without enhancement	Herpes encephalitis, glioma
Mesial temporal sclerosis	Complex partial seizures	Hippocampal atrophy and increased T2/FLAIR. Bilateral in 10 %	Limbic encephalitis, other temporal lobe epileptogenic diseases
Glioma	Insidious course of worsening neurological deficits; new onset seizures; change in mental status	Edema, infiltration and possible enhancement (depending on tumoral grade); possible spread along nerve tracts	Herpes encephalitis, limbic encephalitis, adult type OTC deficiency
Posterior circulation ischemia	Acute visual and memory deficits and altered mental status	Ischemic lesions in PCA territory	Herpes encephalitis
CADASIL^‡^	Recurrent strokes, migraine, cognitive decline	Focal lesions of increased T2 signal in anterior temporal lobe and external capsule. Periventricular and deep white matter lesions throughout the brain; lacunar infarctions	Microangiopathy, cerebral vasculitis
Trauma	History of traumatic insult	Anterior temporal cortical contusions and or epidural or subdural hematomas	Post-traumatic encephalomalacia may simulate atrophy
Radiation necrosis	History of prior radiation to the head and neck	Edema and enhancement within the radiation port	Herpes encephalitis, glioma

^*^HHV-6 – Human Herpes Virus -6

^†^OTC - Ornithine TransCarbamylase

^‡^CADASIL - Cerebral Autosomal Dominant Arteriopathy with Subcortical Infarcts and Leukoencephalopathy
